# Comparative Evaluation of Mechanical Properties, Color Stability, and Surface Roughness of Gingiva‐Shaded Composites

**DOI:** 10.1155/ijod/7735166

**Published:** 2026-09-17

**Authors:** Faris A. Alshahrani, Ibrahim Alsaif, Hassan Alhamza, Abdullah Alhashim, Saleh Alrashed, Noha Taymour, Mohammed M. Gad, Hamad Alrumaih, Yousif A. Al-Dulaijan, Abdulrahman A. Balhaddad

**Affiliations:** ^1^ Department of Substitutive Dental Sciences, College of Dentistry, Imam Abdulrahman Bin Faisal University, P.O. Box 1982, Dammam 31441, Saudi Arabia, iau.edu.sa; ^2^ College of Dentistry, Imam Abdulrahman Bin Faisal University, P.O. Box 1982, Dammam 31441, Saudi Arabia, iau.edu.sa; ^3^ Department of Restorative Dental Sciences, College of Dentistry, Imam Abdulrahman Bin Faisal University, P.O. Box 1982, Dammam 31441, Saudi Arabia, iau.edu.sa

**Keywords:** color stability, immersion solution, mechanical testing, pink composite, surface properties

## Abstract

**Objective:**

This study evaluated the mechanical properties, surface topography, and color stability of a conventional acrylic resin compared to three commercially available gingiva‐shaded composites after immersion in common beverages.

**Methodology:**

Conventional acrylic resin (Major Base 20) was compared with three commercially available pink composites: Shofu Beautifil II (Shofu Inc.), Beauty Gum Gingiva (TuskDent), and Dengen Dent (Dengen Dent). Bar‐shaped specimens (*n* = 10 per material) were tested for flexural strength and elastic modulus using a universal testing machine. Disc‐shaped specimens (*n* = 10 per material) were assessed for Vickers microhardness. Additional disc‐shaped specimens (*n* = 10 per material per solution) were immersed in distilled water, tea, coffee, or Coca‐Cola for 28 days. Color stability (Δ*E*) and surface roughness (Ra) were measured before and after immersion using spectrophotometry and profilometry, respectively. Data were analyzed using one‐way ANOVA and post hoc Tukey’s test (*α* = 0.05).

**Results:**

Shofu Beautifil II (92.24 ± 11.67 MPa) and Beauty Gum (99.28 ± 16.05 MPa) composites exhibited flexural strength comparable to that of acrylic resin (98.49 ± 10.84 MPa). However, Dengen Dent (35.24 ± 5.87 MPa) showed significantly (*p* < 0.001) lower flexural strength compared to the other groups (*p* < 0.001). Both acrylic resin and Dengen Dent had lower elastic modulus and microhardness values than Shofu and Beauty Gum Pink composites (*p* < 0.001). Dengen exhibited significant color changes after immersion in coffee and tea compared to the other groups (*p* < 0.001). In terms of post‐immersion surface roughness, no significant differences were found between the tested groups, except for Shofu Beautifil II, which showed increased roughness when immersed in Coca‐Cola (*p* < 0.05).

**Conclusion:**

Shofu Beautifil II and Beauty Gum Gingiva showed higher flexural strength, elastic modulus, and microhardness, as well as superior color stability (Δ*E*_00_ < 2.8) and minimal surface roughness change compared to Dengen Dent and acrylic resin. Dengen Dent exhibited lower mechanical properties and color instability (Δ*E*_00_ > 2.8), while acrylic resin had moderate performance across all tested parameters.

**Clinical Significance:**

The study underscores the critical need for careful material selection in esthetic dentistry, particularly for gingiva‐shaded composites. Clinicians should be aware that not all pink composites perform equally. Furthermore, the potential for certain composites to develop increased surface roughness when exposed to acidic drinks such as cola may influence plaque retention. Therefore, selecting a pink composite with proven durability, color stability, and surface integrity is essential for achieving predictable and long‐lasting esthetic restorations.

## 1. Introduction

The contemporary landscape of restorative dentistry has shown significant advancements in material science, particularly in the development of esthetic gingival replacement materials that address both functional and cosmetic requirements in prosthetic rehabilitation [1].

Polymethyl methacrylate (PMMA) and other acrylic resins have served as the gold standard for denture base materials and gingival replacement applications due to their favorable mechanical properties, ease of manipulation, and cost effectiveness [2]. The exposure of denture base acrylic resins to acidic media has been shown to alter both surface roughness and hardness properties [3]. Studies examining the influence of various acidic solutions on acrylic resin properties have revealed that even mild acidic conditions can compromise material integrity over time [4, 5], demonstrating the complex relationship between chemical composition and material stability [6, 7]. Furthermore, the degradation mechanisms of acrylic resin materials involve both enzymatic reactions with salivary components and hydrolysis of resin‐containing materials, processes that are accelerated under acidic conditions [8].

The development of specialized pink composites represents a new modality in gingival replacement technology, offering potentially superior esthetic outcomes and enhanced material properties [9]. Recent innovations in pink composite materials feature unique bioactive Giomer Technology [10, 11]. These materials are engineered to provide natural gingival reproduction capabilities while maintaining structural integrity under clinical loading conditions [12]. The availability of multiple shade variations allows for customized color matching across diverse patient populations, addressing a significant limitation of conventional acrylic resin systems [13].

Advanced pink composite formulations extend beyond simple esthetic considerations to incorporate functional benefits that may enhance clinical longevity [14]. They frequently utilize nanofilled hybrid formulations with enhanced physical properties and minimized polymerization shrinkage when compared to conventional alternatives [15]. Despite these advancements, the oral environment presents unique challenges for these materials, with pH fluctuations representing a critical factor in long‐term material performance [16, 17]. Research has consistently demonstrated that acidic environmental conditions can significantly deteriorate the hardness of resin materials while increasing the release of residual monomers, potentially compromising both mechanical properties and biocompatibility [18].

The clinical significance of pH‐related material degradation extends beyond laboratory observations to real‐world performance implications [19]. Aging studies conducted under controlled acidic conditions have shown that denture base materials fabricated using different production methods exhibit varying resistance to pH challenges [20, 21]. Furthermore, the interaction between acidic environments and composite materials involves complex chemical processes that may differ significantly from those observed in traditional acrylic systems, necessitating comprehensive comparative evaluations.

The mechanical performance of dental polymers is governed by compositional and structural factors. The ISO 20795‐1 standard for denture base polymers specifies a minimum flexural strength of 65 MPa [22]. While ISO 4049:2019 applies to polymer‐based restorative materials, it specifies a minimum flexural strength of 50 MPa [23].

Previous studies have demonstrated that composite materials with filler contents exceeding 80% by weight consistently achieve flexural strength results comparable to or exceeding those of conventional acrylic resins [24, 25]. The nanohybrid filler technology employed in both Shofu and Beauty Gum composites creates a three‐dimensional reinforcement network that effectively resists crack propagation under flexural loading [26]. One study reported that flexural strength values of 120–130 MPa for giomer‐based composites, attributed to the prereacted glass ionomer fillers that provide both mechanical reinforcement and chemical bonding to the resin matrix [10]. Materials with prereacted glass ionomer technology exhibit elastic modulus values ranging from 4.0 to 5.5 GPa [27]. However, elevated stiffness concentrates stress at material‐tissue interfaces, predisposing to marginal breakdown, microleakage, or failure in anatomically challenging regions [28]. Hydrophobic resin matrices such as those containing urethane dimethacrylate exhibit low water sorption and resist hydrolytic degradation, preserving mechanical integrity [10].

While the primary clinical applications of PMMA denture bases and esthetic pink gingival composites differ, serving as the foundational structure for removable prostheses and the latter serving as a direct restorative material for gingival esthetics, both are polymeric systems subjected to similar biodegradation challenges in vivo, including chemical, thermal, and mechanical stressors. The objective of this comparison is not to suggest direct interchangeability in clinical practice but rather to evaluate the relative performance characteristics of these materials under simulated biodegradation processes.

While individual properties of pink composites have been reported, there is a paucity of data regarding the simultaneous interplay between mechanical integrity, color stability, and surface degradation of these materials when subjected to acidic and chromatic challenges. Thus, this study aimed to assess the baseline mechanical properties of three commercially available pink composite materials (Shofu Beautifil II, Beauty Gum Gingiva, and Dengen Dent) and investigate their roughness and color stability following controlled immersion in acidic solutions. Given the influence of filler content and resin matrix composition on material performance, it is hypothesized (1) that there would be significant differences in mechanical properties (flexural strength, elastic modulus, and microhardness) among the pink composite materials and the acrylic resin control and (2) that there would be significant differences in color stability and surface roughness among the materials after 28‐day immersion in different staining and acidic beverages.

## 2. Materials and Methods

### 2.1. Ethical Approval

This in vitro study did not involve human participants, animal subjects, or human/animal‐derived data or tissues. IRB approval was exempted by the institutional review board at the College of Dentistry, Imam Abdulrahman Bin Faisal University, Saudi Arabia.

### 2.2. Study Design and Sample Size Calculation

**Figure 1:**
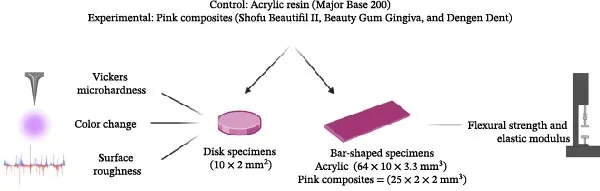
Study design flowchart.

This in vitro investigation compared the properties of conventional acrylic resin with three commercially available pink composites: SHOFU Beautifil II, Beauty Gum Gingiva, and Dengen Dent. The materials employed are detailed in Table 1, and the study flowchart is shown in Figure 1. Mechanical properties assessed included flexural strength, elastic modulus, and Vickers microhardness. Additionally, color change and surface roughness were measured both prior to and following immersion in four distinct acidic solutions; each solution’s ratio, temperature, daily renewal, and pH monitoring followed standardized protocols based on established dental materials literature, with specific brands indicated to support reproducibility [29] Table 2. Sample size determination was performed a priori using G ^∗^Power 3 (Heinrich Heine Universität, Düsseldorf, Germany) to ensure adequate statistical power. The analysis concluded that 10 specimens per group were necessary to detect large effect sizes (*f* = 0.4) with a statistical power of 80% [29].

**Table 1:** Compositional details of materials used in the study.

Group	Material type	Product name	Manufacturer	Resin matrix type	Filler content (%wt)	Filler particle size (μm)	Manufacturing type
1	Control (acrylic resin)	Major Base 20	Major Prodotti Dentari SPA, Moncalieri, Italy	PMMA	N/A^a^	N/A	Heat‐polymerized
2	Experimental pink composite	Shofu Beautifil II	Shofu Dental Corp., San Marcos, CA, USA	Bis‐GMA, UDMA, TEGDMA/Giomer	~83	0.01–5	Light‐cured giomer composite
3	Experimental pink composite	Beauty Gum Gingiva	TuskDent, Moncton, NB, Canada	Bis‐GMA, UDMA	~77	0.01–5	Light‐cured nanohybrid composite
4	Experimental pink composite	Dengen Dent pink composite	Dengen Dental, WY, USA	Bis‐GMA, UDMA, TEGDMA	~73	0.04–3.5	Light‐cured composite resin

^a^Acrylic resin base material is unfilled; details not applicable.

**Table 2:** Beverages and immersion protocol.

Group	Solution type	Product name/brand	Manufacturer/source	Preparation ratio and steps	pH value	Temperature (°C)	Renewal frequency	Storage conditions
1	Control (distilled water)	—	Prepared in Dental Biomaterials Lab	Used as supplied	~7.0	37	Daily	Light‐proof containers, 37°C
2	Tea	Label Tea, Lipton Tea	Unilever, UAE	10 g tea bags in 200 mL boiling water, steeped 5 min, filtered	~5.5	37	Daily	Light‐proof containers, 37°C
3	Coffee	Nescafé Classic	Nestlé, Brazil	10 g instant coffee in 200 mL boiling water, stirred/cool	~5.2	37	Daily	Light‐proof containers, 37°C
4	Cola	Coca‐Cola	The Coca‐Cola Company, Saudi Arabia	Used as supplied	~2.5	37	Daily	Light‐proof containers, 37°C

### 2.3. Specimen Preparation

#### 2.3.1. Flexural Strength and Elastic Modulus Specimens

Bar‐shaped specimens of the pink composites (dimensions: 2 mm × 2 mm × 25 mm; *n* = 10 per group) were prepared in accordance with ISO 4049 standards [23]. The composite resin was placed into a stainless steel mold, covered on both sides with polyester strips and glass slabs, and then light‐cured for 40 s per side using a Satelec Mini LED Curing Light (intensity: 1250 mW/cm^2^; A‐dec Inc., Newberg, OR, USA). Subsequently, the specimens were stored at 37°C for 24 h to complete polymerization.

For the conventional acrylic resin, bar‐shaped specimens measuring 64 mm × 10 mm × 3.3 mm were fabricated via a lost‐wax technique in accordance with ISO 20795‐1:2013 [30]. Wax patterns were invested in metal flasks and subjected to wax elimination to create mold cavities. While the stone mold remained warm, a universal resin separator (Isol Major; Major Prodotti Dentari S.p.A., Moncalieri, Italy) was applied to the mold surfaces to facilitate specimen retrieval. The acrylic resin powder and monomer liquid were mixed according to the manufacturer’s instructions until a dough‐like consistency was achieved. This mixture was packed into the mold cavity, and the flask was sealed and maintained under pneumatic pressure for 30 min. Polymerization was conducted in a thermal curing unit (KaVo Elektrotechnisches Werk GmbH, Leutkirch, Germany) using a two‐stage curing cycle: 73°C for 90 min, followed by 100°C for 30 min. After cooling to room temperature, the specimens were removed from the flasks.

All specimens underwent finishing with a tungsten carbide bur (HM 79GX‐040 HP; Meisinger, Centennial, CO, USA) operating at 18,000 rpm. Dimensional accuracy was verified using a digital caliper (Neiko 01407A Electronic Digital Caliper; Neiko Tools US, LaPorte, IN, USA) with a precision of 0.01 mm.

#### 2.3.2. Microhardness Specimens

Disc‐shaped specimens (10 mm × 2 mm; *n* = 10 per group) were fabricated for both the pink composites and acrylic resin. Pink composite discs were produced using a standardized mold, whereas acrylic resin discs were prepared following the previously described lost‐wax technique. All specimens underwent initial finishing with a tungsten carbide bur and then were sequentially polished under wet conditions employing silicon carbide abrasive papers with grits of 600, 800, 1000, 1200, 2000, and 4000. Specimens exhibiting any internal or external porosities, dimensional inconsistencies, surface defects, warpage, or fractured edges were excluded from further testing to ensure sample integrity.

#### 2.3.3. Color Stability and Surface Roughness Specimens

Disc‐shaped specimens (10 mm × 2 mm; 10 per group per solution) were prepared for both pink composites and acrylic resin using the methods described above. Specimens were divided into four subgroups according to the immersion solution: distilled water (control), tea, coffee, and Coca‐Cola (Table 2). All specimens underwent the same finishing and polishing protocol as the microhardness specimens, and the same exclusion criteria were applied.

#### 2.3.4. Flexural Strength and Elastic Modulus Testing

Flexural strength and elastic modulus were measured using a universal testing machine (INSTRON 5965 load frame; Boston, MA, USA). For the pink composite groups, testing was performed according to ISO 4049 with a 10 mm span at a crosshead speed of 1 mm/min. For the acrylic resin group, testing was conducted according to ISO 20795‐1 using a 50 mm span at a crosshead speed of 5 mm/min. The following formulas were used for calculations:

The flexural strength (*F*) was calculated using the following formula [31]:

F=3LS2WH2,

where *L* = maximum load at fracture (N), *S* = span length (mm), *W* = specimen width (mm), and *H* = specimen height (mm).

The elastic modulus (*M*) was calculated using the following formula [32]:

M=LS334WH3d,

where *M* = elastic modulus, *L* = maximum load (N), *S* = span length (mm), *W* = specimen width (mm), *H* = specimen height (mm), and *d* = defluxion corresponding to the load (*L*).

#### 2.3.5. Microhardness Evaluation

Vickers microhardness was measured using a microhardness tester (Tukon 1102 Wilson Microhardness Tester; Buehler, Germany) with a 50 g load applied for 10 s. Three indentations were made on each specimen, and the average value was recorded. The Vickers’ hardness number (VHN) was calculated using the formula [31]:

VHN=1854.4×Ld2,

where *L* = load (gf) and *d* = mean diagonal length (μm).

#### 2.3.6. Surface Roughness Analysis

Surface roughness (Ra, μm) was assessed at baseline and after 28 days of immersion using a noncontact profilometer (Contour GT‐K1 optical profiler; Bruker Nano, Tucson, AZ, USA). To ensure consistent measurement locations, a custom putty holder with immovable reference marks was fabricated. Three different sites were scanned on each specimen, and the average values of Ra (arithmetic mean deviation), Rv (maximum valley depth), and Rt (maximum height of the profile) were recorded. The changes in roughness parameters were calculated by subtracting the baseline values from the post‐immersion values [29].

#### 2.3.7. Color Stability Assessment

In order to evaluate the color change, an initial recording of the specimens’ color was carried out (*T*_0_). The specimens’ color coordinates were then measured at *T*_1_ (after 28 days) using a spectrophotometer (Color‐Eye 7000A; X‐rite, Grand Rapids, MI). Following manufacturer recommendations, calibration was achieved using white and black ceramic tiles. Using the color system CIE *L* ^∗^ *a* ^∗^ *b* ^∗^, the color values were measured three times per specimen. Solutions were replaced daily to maintain consistency, with all preparations being standardized by a single operator. The CIEDE2000 color difference (Δ*E*_00_) was calculated as described and detailed in a previous study [33].

ΔE=ΔL′KLSL2+ΔC′KCSC2ΔH′KHSH2+RT ΔC′KCSC2 ΔH′KHSH,

where Δ*L*′, $\Delta C_{ab}^{\prime }$, and $\Delta H_{ab}^{\prime }$ represent differences in lightness, chroma, and hue, respectively; *S*_*L*_, *S*_*C*_, and *S*_*H*_ are weighting functions; *K*_*L*_, *K*_*C*_, and *K*_*H*_ are parametric factors; and *R*_*T*_ is a rotation function.

### 2.4. Statistical Analysis

Descriptive statistics (mean, standard deviation, frequency, and percentages) were used to summarize the information. The normality test was checked using the Shapiro–Wilk test. For microhardness, a one‐way ANOVA was used to compare the four material groups. For color change and surface roughness, a two‐way ANOVA was used to evaluate the main effects (material and immersion solution) and their interaction, followed by Tukey’s HSD post hoc test for pairwise comparisons. For flexural strength and elastic modulus, because the acrylic resin specimens were of different dimensions and tested under a different ISO standard, statistical comparisons were performed only among the three pink composites using one‐way ANOVA and Tukey’s test; the acrylic resin data are presented descriptively. A 5% significance level was used in all tests. The data were analyzed using Sigma Plot 12.0 (SYSTAT, Chicago, IL, USA).

## 3. Results

**Figure 2:**
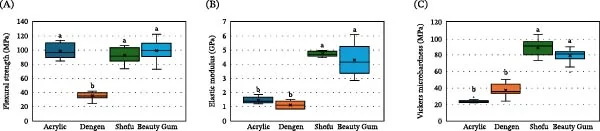
The mechanical properties of resin‐based pink composites. (A) Flexural strength, (B) elastic modulus, and (C) Vickers microhardness. Different letters indicate a statistically significant difference (*p* < 0.05).

Among the pink composites, Shofu Beautifil II (92.24 ± 11.67 MPa) and Beauty Gum Gingiva (99.28 ± 16.05 MPa) exhibited flexural strength (Figure 2A) that was significantly higher than that of Dengen Dent (35.24 ± 5.87 MPa; *p* = 0.0003, power = 100%). The acrylic resin, tested under ISO 20795‐1 conditions, yielded a mean flexural strength of 98.50 ± 10.84 MPa, which is reported separately because of the different specimen geometry and test configuration. For the elastic modulus (Figure 2B), the acrylic resin (1.46 ± 0.24 GPa) and Dengen (1.11 ± 0.29 GPa) demonstrated lower values (*p* < 0.05) than Shofu (4.74 ± 0.18 GPa) and Beauty Gum (4.28 ± 1.17 GPa). However, the elastic modulus of the acrylic resin was obtained from specimens with different dimensions and span; therefore, only the composites were compared statistically. For Vickers microhardness (Figure 2C), also acrylic resin (21.87 ± 1.94 VHN) and Dengen (37.31 ± 8.20 VHN) demonstrated lower values (*p* = 0.001; power of analysis = 100%) than Shofu (88.78 ± 9.15 VHN) and Beauty Gum (78.70 ± 8.73 VHN).

**Figure 3:**
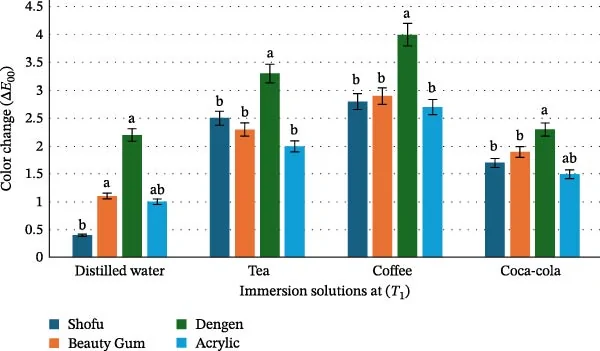
The color change (Δ*E*) of the resin‐based pink composites after immersion in different solutions for 28 days. Different letters indicate a statistically significant difference (*p* < 0.05).

**Figure 4:**
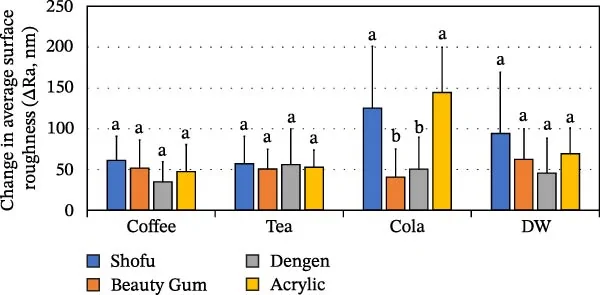
The average surface roughness (Ra, µm) of the resin‐based pink composites after immersion in different solutions for 28 days. Different letters indicate a statistically significant difference (*p* < 0.05).

**Figure 5:**
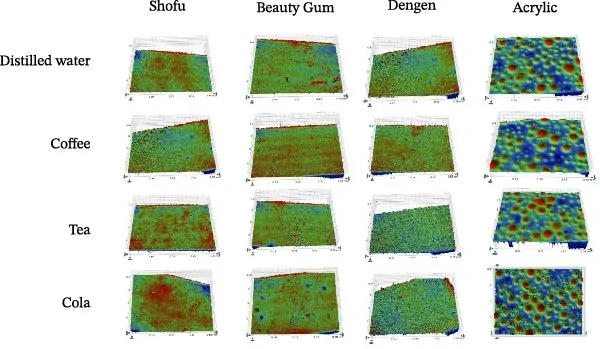
Representative profilometer images depicting the surface topography of all tested material groups following immersion in different solutions for 28 days.

In Figure 3, Dengen exhibited significant color changes after immersion in coffee (Δ*E*_00_ = 4) and tea (Δ*E*_00_ = 3.3) compared to the other groups (*p* < 0.001; power of analysis = 100%). All groups demonstrated clinically acceptable color changes (Δ*E*_00_ < 2.8) after immersion in Coca‐Cola and distilled water (*p* > 0.05). Figure 4 displays the post‐immersion surface roughness changes (nm). No significant differences were observed between the tested groups, except for Shofu (125.29 ± 75.88), which displayed increased surface roughness when immersed in Coca‐Cola compared to the other pink composites (*p* = 0.019; power of analysis ≥ 80%). Representative profilometric images of the surface topography for all tested materials, Shofu Beautifil II, Beauty Gum Gingiva, Dengen Dent composite, and conventional acrylic resin, after 28 days of immersion in distilled water, tea, coffee, and Coca‐Cola, are presented in Figure 5. The images reveal overall smooth surface profiles for Shofu and Beauty Gum, correlating with their lower Ra values. Dengen exhibited greater surface irregularities following immersion in tea and coffee. Acrylic resin exhibited localized crater‐like topographical features, likely attributable to porosity from monomer evaporation during processing; however, these localized defects did not significantly elevate the overall arithmetic average roughness (Ra). Shofu showed subtle but measurable surface roughening after Coca‐Cola immersion, aligning with the quantitative increases in roughness recorded.

## 4. Discussion

The present in vitro study assessed the mechanical properties and surface characteristics of conventional acrylic resin and three commercially available pink composites (Shofu Beautifil II, Beauty Gum Gingiva, and Dengen Dent) before and after immersion in various solutions. The findings revealed that, among the pink composites, Shofu and Beauty Gum exhibited significantly higher flexural strength than Dengen (*p* < 0.05). Microhardness was significantly greater for Shofu and Beauty Gum than for acrylic resin and Dengen (*p* < 0.05). Dengen showed pronounced color changes in coffee and tea (*p* < 0.001). In terms of surface roughness, no significant intergroup differences emerged post‐immersion, except for Shofu in Coca‐Cola (*p* < 0.05). Thus, the research hypotheses were partially accepted.

The flexural strength performance of Shofu Beautifil II (92.24 ± 11.67 MPa) and Beauty Gum Gingiva (99.28 ± 16.05 MPa) is comparable to that of conventional acrylic resin (98.50 ± 10.84 MPa). These values align closely with the ISO 20795‐1:2013 standard for denture base polymers and the ISO 4049:2019 standard for polymer‐based restorative materials [23, 30]. A direct comparison of flexural strength between the composites and acrylic resin is precluded because the two material classes were tested using different specimen geometries and span lengths, as mandated by their respective ISO standards. Therefore, the following discussion of flexural properties focuses on the pink composites.

Previous studies have demonstrated that composite materials with filler contents exceeding 80% by weight consistently achieve flexural strength results comparable to or exceeding those of conventional acrylic resins [24, 25]. The superior performance of Shofu and Beauty Gum is likely attributed to their high filler content and nanohybrid filler technology, which creates a three‐dimensional reinforcement network resisting crack propagation [26] and, in the case of Shofu, prereacted glass ionomer fillers that provide chemical bonding to the resin matrix [10]. Conversely, the dramatically reduced flexural strength of Dengen Dent (35.24 ± 5.87 MPa) represents a critical performance deficit that significantly limits its clinical applicability. This value falls well below the minimum threshold established for dental prosthetic materials and suggests fundamental formulation inadequacies [22].

The substantial strength reduction may be attributed to inadequate filler content, poor silanization of filler particles, or incomplete polymerization during manufacturing [34]. Similar findings have been reported for low‐quality composite materials, where insufficient filler loading or poor interfacial bonding result in premature failure under stress [35].

The elastic modulus results of Shofu (4.74 ± 0.18 GPa) and Beauty Gum (4.28 ± 1.17 GPa) demonstrate significantly higher stiffness compared to acrylic resin (1.46 ± 0.24 GPa) and Dengen (1.11 ± 0.29 GPa). The higher elastic modulus of Shofu and Beauty Gum indicates superior resistance to deformation under load, attributed to their high‐density filler networks and cross‐linked resin matrices [22, 36]. However, elevated stiffness may concentrate stress at material‐tissue interfaces, potentially predisposing to marginal breakdown in anatomically challenging regions [28]. This aligns with a previous study demonstrating that materials with prereacted glass ionomer technology exhibit elastic modulus values ranging from 4.0 to 5.5 GPa [27]. Another possible explanation is that both Shofu and Beauty Gum employ hydrophobic resins (urethane dimethacrylate) with low water sorption, minimizing plasticization effects, aligning with a previous study showing that HEMA‐free matrices resist hydrolytic degradation, preserving mechanical integrity [10].

The lower elastic modulus values observed in the Dengen composite suggest an increased risk of deformation and wear under functional loading conditions, which must be balanced against potential benefits in accommodating thermal expansion [28].

The microhardness results demonstrate a clear contrast in material performance, with Shofu (88.78 ± 9.15 VHN) and Beauty Gum (78.70 ± 8.73 VHN) significantly exceeding both acrylic resin (21.87 ± 1.94 VHN) and Dengen (37.31 ± 8.20 VHN). These findings are consistent with a previous study demonstrating that highly filled composite materials exhibit superior surface hardness compared to that of conventional polymers [37]. The enhanced microhardness of Shofu and Beauty Gum composites suggests a potentially improved resistance to wear and surface degradation in vitro, which may influence the maintenance of esthetic outcomes attributed to their nanohybrid filler systems creating a dense, highly crosslinked surface layer [38] and surface mineralization reactions from bioactive fillers, which further enhance hardness [39]. Studies have shown that composites with optimized filler size distribution and surface treatment achieve microhardness values exceeding 70 VHN, correlating with improved clinical durability [40]. The relatively poor performance of Dengen may arise from monomer leaching or incomplete polymerization, while conventional acrylic resin’s lower hardness is likely due to its susceptibility to chemical softening in acidic environments [5].

The color stability analysis reveals critical differences in material performance that directly impact clinical esthetics. The Dengen composite demonstrated unacceptable color changes in coffee (Δ*E*_00_ = 4) and tea (Δ*E*_00_ = 3.3), both exceeding the clinically acceptable perceptibility threshold of (Δ*E* = 2.8), indicating pronounced staining potential. This is likely due to poor matrix polymerization and high porosity, allowing chromogenic substances to penetrate and accumulate within the material structure [41]. In contrast, Shofu and Beauty Gum composites demonstrated color changes below or near the perceptibility threshold across all tested solutions, including coffee and tea. This resistance is attributed to their hydrophobic resin matrices and dense filler networks, which minimize water sorption and stain penetration [10]. Interestingly, all materials demonstrated acceptable color stability in Coca‐Cola and distilled water (Δ*E* < 4), suggesting that the staining mechanism is primarily related to polyphenolic compounds in coffee and tea rather than acidity alone. This finding aligns with previous research demonstrating that tannins and chromogenic compounds in beverages are the primary contributors to composite staining [42].

The surface roughness analysis as shown in Figure 5 revealed minimal changes across most material‐solution combinations, with the notable exception of Shofu in Coca‐Cola (125.29 ± 75.88 nm). This finding suggests that surface topography changes are primarily material‐specific rather than solution‐dependent. The increased roughness observed with Shofu in Coca‐Cola may be attributed to the interaction between phosphoric acid and the bioactive glass ionomer fillers, leading to selective dissolution of surface components [43]. Studies have demonstrated that acids with low pH, especially those containing phosphoric acid, induce selective leaching and degradation of glass ionomer fillers, resulting in morphological alterations and increased surface roughness. The process involves ion‐exchange reactions that compromise filler‐matrix interfaces, thus facilitating the emergence of micro‐porosities and valleys in the material surface [33, 39]. It is important to note that even after such exposure, the roughness values observed in Shofu remain below the 200 nm clinical threshold generally considered acceptable for minimizing plaque retention and bacterial adherence [44]. Regarding the acrylic resin, profilometric images (Figure 5) revealed localized crater‐like topographical features, likely resulting from porosity due to monomer evaporation during processing. Despite these qualitative visual observations, the overall arithmetic average roughness (Ra) remained low as the intercrater matrix remained highly polished. For load‐bearing applications, the superior flexural strength and elastic modulus of premium pink composites suggest potential advantages in stress distribution and fatigue resistance. The enhanced microhardness values indicate improved wear resistance in vitro, a critical factor in preserving esthetic outcomes. Beyond statistical significance, the observed differences meet or exceed clinical thresholds for flexural strength, color stability, and surface roughness in dental materials. Large effect sizes confirm that the variations are not only statistically reliable but also meaningful in everyday clinical application [45], as shown in Table 3. The findings support preferential use of Shofu and Beauty Gum in prosthetic and restorative procedures demanding durability and esthetic reliability while highlighting the clinical limitations of Dengen composite.

**Table 3:** Summary for the effect size and clinical threshold of the tested materials.

Parameter	Significant?	Effect size (partial *η* ^2^)	Clinical threshold	Clinical interpretation
Flexural strength	Yes	0.44	≥65 MPa	Dengen unacceptable
Elastic modulus	Yes	0.38	High modulus: load resistance	Shofu/Beauty Gum optimal
Microhardness	Yes	0.47	>70 VHN	Shofu/Beauty Gum durable
Color stability	Yes	0.52	Δ*E*_00_ < 2.8	Dengen prone to staining
Surface roughness	Yes (Shofu/Cola)	0.29	<200 nm	Within acceptable range

The present study has some limitations that should be acknowledged. First, a direct statistical comparison of flexural strength and elastic modulus between the acrylic resin and the pink composites is precluded due to differing ISO‐mandated specimen geometries and span lengths, which represents a limitation when interpreting the comparative mechanical integrity of these material classes. Second, the study focused on short‐term exposure to acidic solutions, which may not reflect the cumulative effects of prolonged clinical service. Additionally, this in vitro design lacked simulation of oral aging factors, such as mechanical fatigue, biofilm formation, pH cycling, and artificial saliva. Extended aging studies incorporating these parameters, alongside the investigation of repair protocols, would provide more comprehensive performance data.

## 5. Conclusions

Within the limitations of this in vitro study, Shofu Beautifil II and Beauty Gum Gingiva demonstrated significantly higher flexural strength, elastic modulus, and microhardness than Dengen Dent, with mechanical properties comparable to those of conventional acrylic resin. Dengen Dent exhibited clinically unacceptable color instability (Δ*E*_00_ > 2.8) in tea and coffee, whereas Shofu and Beauty Gum maintained color stability across all solutions. Although surface roughness remained below the 200 nm clinical threshold for all materials, Shofu showed a statistically significant increase in roughness after Coca‐Cola immersion. Direct comparison between acrylic resin and composites was limited by differing ISO test geometries.

## Author Contributions

Conceptualization: Faris A. Alshahrani and Abdulrahman A. Balhaddad. Methodology: Ibrahim Alsaif, Saleh Alrashed, and Noha Taymour. Software: Hassan Alhamza. Validation: Ibrahim Alsaif, Hassan Alhamza, Abdullah Alhashim, and Saleh Alrashed. Formal analysis, supervision: Mohammed M. Gad. Investigation: Hamad Alrumaih. Resources: Noha Taymour. Data curation: Abdulrahman A. Balhaddad. Writing – original draft preparation: Noha Taymour, Faris A. Alshahrani, and Yousif A. Al‐Dulaijan. Writing – review and editing: Noha Taymour, Yousif A. Al‐Dulaijan, and Abdulrahman A. Balhaddad. Visualization: Yousif A. Al‐Dulaijan. Project administration: Hamad Alrumaih and Faris A. Alshahrani.

## Funding

This study did not receive any specific funding.

## Disclosure

All authors have read and approved the final version of the manuscript. All authors have full access to all of the data in this study and took complete responsibility for the integrity of the data and the accuracy of the data analysis.

## Ethics Statement

Ethical approval was not required as this in vitro study did not involve human participants, animal subjects, or human/animal‐derived data or tissues.

## Conflicts of Interest

The authors declare no conflicts of interest.

## Data Availability

The authors confirm that the data supporting the findings of this study are available upon request from the authors.
